# QoS and Energy Aware Cooperative Routing Protocol for Wildfire Monitoring Wireless Sensor Networks

**DOI:** 10.1155/2013/437926

**Published:** 2013-06-13

**Authors:** Mohamed Maalej, Sofiane Cherif, Hichem Besbes

**Affiliations:** Engineering School of Telecommunications of Tunis (Sup'Com), City of Communications Technologies, El Ghazala 2083, Ariana, Tunisia

## Abstract

Wireless sensor networks (WSN) are presented as proper solution for wildfire monitoring. However, this application requires a design of WSN taking into account the network lifetime and the shadowing effect generated by the trees in the forest environment. Cooperative communication is a promising solution for WSN which uses, at each hop, the resources of multiple nodes to transmit its data. Thus, by sharing resources between nodes, the transmission quality is enhanced. In this paper, we use the technique of reinforcement learning by opponent modeling, optimizing a cooperative communication protocol based on RSSI and node energy consumption in a competitive context (RSSI/energy-CC), that is, an energy and quality-of-service aware-based cooperative communication routing protocol. Simulation results show that the proposed algorithm performs well in terms of network lifetime, packet delay, and energy consumption.

## 1. Introduction

  The automatic monitoring of wildfire generally supports multimodal observations. This is due to the extent of  the areas to be covered and the difficulty of detecting fire. In fact, most fire detection techniques, for example, based on the video, suffer from false alarms. The use of wireless sensor networks (WSNs) can improve the quality of the detection and consequently the reduction of the false alarm. WSN can be easily deployed and do not require special auxiliary installation. They are mainly used to control buildings, houses, or archaeological sites in the forest.

However, the forest environment presents the problem of wide covered areas requiring the transmission of a large amount of information through the network with the risk of significant energy consumption and hence limiting the lifetime of the network. Particularly, energy parameter is crucial for the wildfire application. This is due to the complexity of maintenance of the sensors and the substitution of dead batteries due to the difficulty of access to these sensors placed generally in large covered areas. The second problem which arises in this type of environment is the fading effect due to the presence of trees leading to an important shadowing phenomenon.

To solve these problems, we propose a new methodology to design and optimize WSN based on both energy conservation and consideration of the quality of transmission for choosing the routing protocol.

Cooperative communication is a promising solution for enhancing WSN lifetime. In recent works, this concept has been proposed to exploit the spatial diversity gains in wireless networks [1–3]. Data aggregation in WSN often uses multihop transmission techniques. At each hop, the network relies on only one sensor. This often results in a significant decrease in the energy of some sensors and thus limits the lifetime of the network while a large number of sensors are still in working condition. The main idea of cooperative communication consists in relying, at each hop, on the resources of multiple nodes or relays (called cooperative nodes) to transmit data from one sensor to another, instead of using only one sensor as relay. Thus, by sharing resources between nodes, the transmission quality is enhanced.

It is also obvious that the use of a cooperative scheme improves the reliability of communication in case of fire propagation. Indeed, the presence of several relays for each possible hop ensures the further communication of information and therefore the possibility of detection and tracking of potential wildfire.

Thus, cooperative mechanism is the key to the performance of cooperative communication protocols. However, it is challenging to find the optimal cooperative policies in dynamic WSN, where reinforcement learning (RL) algorithms can be used to find the optimal control policy without the need of centralized control.

Recently, a cooperative communication protocol for quality-of-service (QoS) provisioning has been proposed and named MRL-CC, a multiagent reinforcement learning-based cooperative communication routing algorithm [1]. The RL concept consists in considering the cooperative nodes as multiple agents learning their optimal policy through experiences and rewards. MRL-CC has been based on internode distance and packet delay to enhance the QoS metrics. However, it does not care about energy consumption and network lifetime which are important components for energy efficiency.

In this paper, we design cooperative communication routing protocol based on both energy consumption and QoS. The QoS is measured by the absolute received signal strength indicator (RSSI). To integrate these two parameters in the routing protocol, we use a competitive/opponent mechanism implemented at each node by the multiagent reinforcement-learning (MRL) algorithm. Our proposed algorithm (RSSI/energy-CC) is also an energy and QoS aware routing protocol since it ensures better performance in terms of end-to-end delay and packet loss rate, taking into account the consumed energy through the network.

The rest of the paper is organized as follows.  Section 2 describes the RL algorithm and the design and implementation of MRL-CC algorithm and our algorithm, the RSSI/energy-CC. The performance analysis is presented in Section 3. Finally, Section 4 concludes the paper and gives future research discussions.

## 2. Cooperative Communication in WSN Using Reinforcement Learning

In this section, the background information on RL is provided. Then, we give an overview about the architecture and design issues of our concept of cooperative communication in WSN. Then, we describe the architecture and design issues of MRL-CC, a cooperative communication algorithm using RL. After that, we explain the architecture of new algorithm, RSSI/energy-CC, taking into account both QoS and energy consumption.

### 2.1. Reinforcement Learning

**Figure 1:**
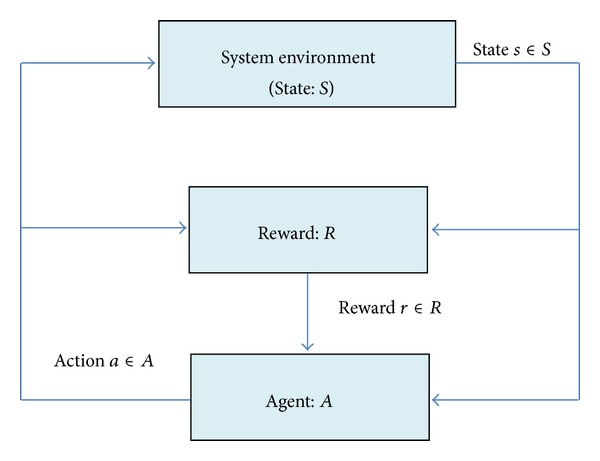
Reinforcement learning model.

RL provides a framework in which an agent can learn control policies based on experiences and rewards. In the standard RL model, an agent is connected to its environment via perception and action, as shown in Figure 1. On each step of interaction, the agent receives as an input, *i*, some indication of the current state, *s*, of the environment; the agent then chooses an action, *a*, to generate as an output. The action changes the state of the environment, and the value of the state transition is communicated to the agent through a scalar RL signal, *r*. Depending on its behavior, the agent should choose actions that tend to increase the long-term sum of values of the reinforcement signal [4].

The main idea of  RL is to strengthen the good behaviors of the agent while weakening the bad behaviors through rewards given by the environment.

The environment of the agent is described by a Markov decision process (MDP). An MDP models an agent acting in an environment with a tuple (*S*, *A*, *P*, *R*), where *S* is a set of states and *A* denotes a set of actions. *P*(*s*′ | *s*, *a*) is the transition model that describes the probability of entering state *s*′ ∈ *S* after executing action *a* ∈ *A* at state *s* ∈ *S*. *R*(*s*, *a*, *s*′) is the reward obtained when the agent executes *a* at *s* and enter *s*′. The goal of solving an MDP is to find an optimal policy, *π* : *S* ↦ *A*, that maps states to actions such that the cumulative reward is maximized [4].

Multiagent systems (MASs) are systems showing that multiple agents are connected to the environment and that they may take actions to change the state of the environment. The generalization of the Markov decision process to the multiagent case is the stochastic game (SG) [5].

In MAS case, each agent assumes itself as the only one that can change the state of the environment and does not consider the interactions between itself and other agents. Therefore, the state transitions are the result of the joint action of all agents, **a** = [*a*_1_,…, *a*_*n*_], where *n*is the number of agents. Consequently, the rewards for each agent *R*_*i*_,  *i* = 1,…, *n*, also depends on the joint action. The policies *π*_*i*_ : *S* ↦ *A* form together the joint policy Π.

If *R*_1_ = ⋯ = *R*_*n*_, all the agents have the same goal (to maximize the same expected return), and the SG is fully cooperative. If *n* = 2 and *R*_1_ = −*R*_2_, the two agents have opposite goals, and the SG is fully competitive. Mixed games are stochastic games that are neither fully cooperative nor fully competitive.

### 2.2. Cooperative Communication Concept in WSN

#### 2.2.1. Adopted Architecture

**Figure 2:**
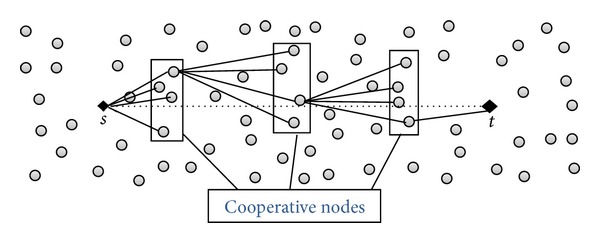
Multihop mesh cooperative structure for data dissemination in WSNs.

For reliable data dissemination in WSNs, we use a multihop mesh cooperative structure. It consists in forming groups of cooperative nodes (denoted as CN) between the source node and the sink node. The data packets originated from a source node are forwarded towards the sink by these CN groups (Figure 2) using a multihop transmission. When a data packet is received by a CN group, a node from that group will be elected to broadcast the data packet to the adjacent CN group. The other nodes of that CN group will help in the packet forwarding in case the elected node fails in data packet transmission or in case the packet is corrupted.

**Figure 3:**
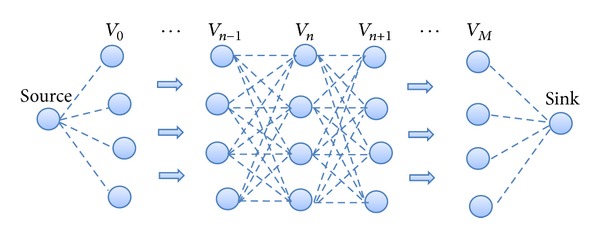
Cooperation between adjacent groups of cooperative nodes.

Therefore, we can show the group of nodes connected to each other in a multihop mesh cooperative structure in Figure 3. In fact, the set of *n*th cooperative group (denoted by *V*_*n*_) is connected with *V*_*n*−1_ and *V*_*n*+1_, which are one hop farther and closer towards the sink than *V*_*n*_, respectively, that is, each node in *V*_*n*_ is connected with all nodes in *V*_*n*−1_ and *V*_*n*+1_.

To construct a multihop mesh cooperative structure, a set of nodes, termed as reference nodes (denoted as RN), between the source node and the sink node is first selected. After that, a set of nodes around each RN will be selected as CN, and thus a multihop mesh cooperative structure is constructed in this phase [6].

#### 2.2.2. WSN Modeling with RL

From the point of view of RL, we can consider a WSN as multiagent system. In fact, sensor nodes can be considered as agents interacting with the environment which can be represented for node *i* ∈ *V*_*n*_ as follows.

 (i) *State*: the CN groups are modeled to be the environment states:
  (1) $$\begin{array}{c} s_{n}=\left\{k\right\},\;\text{where}\;\;k\in \left\{\ldots ,V_{n-1},V_{n},V_{n+1},\ldots \right\}. \end{array}$$
 (ii) *Action*: an agent can operate one of these two actions:
   
*a*_*f*_: forwarding of the packet from *V*_*n*_ to *V*_*n*+1_,
   
*a*_*m*_: monitoring the forwarded packet;
   so: *A* = {*a*_*f*_, *a*_*m*_}.

In our study, we have considered two approaches. The first approach is proposed in [1] where the RL strategy (policy, behaviors, and rewards) for the sensor nodes considers the packet delay and the packet loss rate. This technique has been called the MRL-CC algorithm. The goal of MRL-CC is to enhance packet delay and packet loss rate. The second approach is treated in our work in [7] where the RL strategy is based on the link quality between sensor nodes and their amount of energy consumption. Our strategy goal is to enhance energy efficiency and lifetime of the WSN, that is, to reduce network energy consumption and to maximize network lifetime.

### 2.3. Multiagent Reinforcement Learning-Based Cooperative Communication Routing Algorithm (MRL-CC) 

#### 2.3.1. MRL-CC Implementation

Node election in the CN group is based on a multiagent RL algorithm, performing a fully cooperative task using a “*Q*-learning” algorithm. The strategy is described as follows.

 (i) *Behavior*: each node maintains *Q*-values of itself and its cooperative partners which reflect the qualities (transmission delay, packet delivery ratio) of the available routes to the sink.
 (ii) *Policy*: when a packet is received by the nodes in a CN group, each node will compare its own *Q*-value with those of other nodes in the CN group; the node which determines that it has the highest *Q*-value will be elected to forward the data packet to the adjacent CN group towards the sink. The other cooperative nodes will monitor the packet transmission at the next hop.
 (iii) *Reward*: the reward function is defined as follows:

(2a) ri=((dVn,sink−dVn+1,sink)/dVn,sink)((TVn+1−TVn)/Trmn),

(2b) ri=−TrfTrmn.

Equation (2a) is used to calculate the reward when the packet forwarding is successful, where *d*_*V*_*n*_,sink_ is the average distance between *V*_*n*_ and the sink, which can be calculated as

(3) $$\begin{array}{c} d_{V_{n},\text{sink}}=\frac{1}{N_{V_{n}}}\underset{i\in V_{n}}{\sum }d_{i,\text{sink}} \end{array}$$

where *N*_*V*_*n*__ is the number of cooperative nodes in *V*_*n*_, *T*_*V*_*n*+1__ and *T*_*V*_*n*__ are the packet forwarding time at *V*_*n*+1_ and *V*_*n*_, respectively; *T*_*rmn*_ is the maximum amount of time that can be elapsed in the remaining path to the sink to meet the QoS requirements on end-to-end delay. The positive reward reflects the quality of the packet forwarding.

Equation (2b) is used to calculate the reward when the packet forwarding fails; *T*_*rf*_ is the packet reforwarding timer used for failed forwarding packets. The negative reward reflects the delay caused by the unsuccessful packet transmission from *V*_*n*_ to *V*_*n*+1_.

 (i) *Q-value update*: in MRL-CC, for 1-hop forwarding, at iteration *t*, node *i* ∈ *V*_*n*_ forwards a packet to *V*_*n*+1_, and then *j* ∈ *V*_*n*+1_ is elected to continue packet forwarding. Therefore, node *i* updates its *Q*-value as
  (4) Qit+1(sit,ait) =(1−α)Qit(sit,ait)  +α(rit+1(sit+1)+γω(i,j)max⁡aj∈AQjt(sjt,ajt)      +γ∑i′∈Vni′≠iω(i,i′)max⁡ai′∈AQi′t(si′t,ai′t)),

where *γ* ∈ [0,1] is the discount factor, *α* ∈ [0,1] is the learning rate parameter and *ω*(*i*, *j*) and *ω*(*i*, *i*′) are, respectively, factors that weigh the maximum *Q*-value for node *j* in *V*_*n*+1_ and the maximum *Q*-value of node *i*′ (neighbor of node *i*) in *V*_*n*_.

Equation (4) shows that the *Q*-value of node *i* is a weighed sum of the *Q*-value of node *i* at the previous state, the action's immediate reward, the maximum *Q*-value of *j* which is elected as the forwarding node in *V*_*n*+1_ at the next hop, and the *Q*-values of all of *i*'s cooperative partners in *V*_*n*_.

Note that in the initialization phase, each node is assigned with an initial *Q*-value. For node *i* ∈ *V*_*n*_, its initial *Q*-value (denoted as *Q*_*i*_^ini^) is calculated based on the relative distance (compared with its cooperative partners in *V*_*n*_) from node *i* to the nodes in *V*_*n*+1_, as shown in the following:

(5) $$\begin{array}{c} Q_{i}^{\text{ini}}=\frac{d_{V_{n},V_{n+1}}}{d_{i,V_{n+1}}}, \end{array}$$

where *d*_*V*_*n*_,*V*_*n*+1__ is the average distance between *V*_*n*_ and *V*_*n*+1_, which can be calculated as

(6) $$\begin{array}{c} d_{V_{n},V_{n+1}}=\frac{1}{N_{V_{n}}}\underset{i\in V_{n}}{\sum }d_{i,V_{n+1}}, \end{array}$$

where *N*_*V*_*n*__ is the number of cooperative nodes in *V*_*n*_.

The average distance between node *i* and *V*_*n*+1_, denoted by *d*_*i*,*V*_*n*+1__, can be calculated as

(7) $$\begin{array}{c} d_{i,V_{n+1}}=\frac{1}{N_{V_{n+1}}}\underset{j\in V_{n+1}}{\sum }d_{i,j}. \end{array}$$

#### 2.3.2. Interpretation

We can conclude that MRL-CC algorithm is considering each CN group as one single node because it is performing a fully cooperative task. In fact, all nodes of one CN group get the same positive/negative reward after each transmission procedure. The value of that reward represents the quality of packet forwarding in terms of delay and packet loss rate. Besides, the *Q*-values of the cooperative nodes are initially based on average distance. Therefore, by electing a node with the highest *Q*-value, we also understand that the policy adopted in MRL-CC is based on node election with the shortest distance and the lowest packet delay. Thus, MRL-CC ensures communication reliability. However, it has no information about energy consumption that can be a useful parameter to be considered in RL.

### 2.4. WSN Modeling with Reinforcement Learning in RSSI/Energy-CC Algorithm

#### 2.4.1. Main Idea

Nodes in a CN group will be considered as opponents to each other, so that, each node will maintain a *Q*-value which reflects the payoff that would have been received if that node selected the action *a*_*f*_ and the other nodes jointly selected the action *a*_*m*_. After that, the node with the highest total payoff will be elected to forward the data packet to the next CN group towards the sink.

For the rewarding procedure, there are two cases.

 (i) Transmission succeeded: the *Q*-values of each node will be updated according to its energy consumption compared to its neighbors in its CN group.
 (ii) Transmission failed: the *Q*-value of the node that failed to forward the data packet will be updated with a negative reward, whereas for the other nodes, their *Q*-value will be updated according to an indication about their signal quality.

In our work, we have chosen to use the RSSI as an available indication about signal quality for each packet received at a sensor node.

#### 2.4.2. RSSI/Energy-CC Algorithm Strategy

Node election in the CN group is based on a multiagent RL algorithm, performing a fully competitive task using an “opponent modeling” algorithm [8]. The strategy is described as follows.

 (i) *Policy*: node election, for packet forwarding, for the node with the best link quality and the lowest energy consumption, or a tradeoff between the two criteria.
 (ii) *Behavior*: each node maintains *Q-*values which reflects the payoff that would have been received if that node selected the forwarding action *a*_*f*_ and another node in its CN group selected the monitoring action *a*_*m*_.
 (iii) *Reward*: Each time a packet is forwarded, all the nodes will receive immediate rewards from the environment, which represent a tradeoff about energy consumption and quality of the received signal.

#### 2.4.3. Algorithm Initialization Phase

In the initialization phase, each node is assigned with an initial value regarding its opponents in *V*_*n*_. The initial payoff of node *i* ∈ *V*_*n*_ compared to its neighbor *i*′ is the *Q*-value calculated based on its absolute RSSI in dBm measured from the next cooperative group *V*_*n*+1_. The *Q*-value is defined as follows:

(8) $$\begin{array}{c} Q_{i^{\prime },\;i}^{\text{ini}}=\frac{\text{RSS}{\text{I}}_{i,V_{n+1}}-\text{RSS}{\text{I}}_{i^{\prime },V_{n+1}}}{\text{RSS}{\text{I}}_{V_{n},V_{n+1}}}, \end{array}$$

where RSSI_*V*_*n*_,*V*_*n*+1__ is the average RSSI between *V*_*n*_ and *V*_*n*+1_, which can be calculated as

(9) $$\begin{array}{c} \text{RSS}{\text{I}}_{V_{n},V_{n+1}}=\frac{1}{N_{V_{n}}}\underset{i\in V_{n}}{\sum }\text{RSS}{\text{I}}_{i,V_{n+1}}, \end{array}$$

where *N*_*V*_*n*__ is the number of cooperative nodes in *V*_*n*_.

The average RSSI between node *i* and *V*_*n*+1_, RSSI_*i*,*V*_*n*+1__,  can be calculated as

(10) $$\begin{array}{c} \text{RSS}{\text{I}}_{i,V_{n+1}}=\frac{1}{N_{V_{n+1}}}\underset{j\in V_{n+1}}{\sum }\text{RSS}{\text{I}}_{i,j}. \end{array}$$

#### 2.4.4. Data Dissemination Phase

When a data packet is received by a CN group *V*_*n*_, each node will compare its own total payoff, regarding all its opponents, with those of other cooperative nodes.

The node which determines that it has the highest total payoff will forward the data packet to *V*_*n*+1_, and other nodes in *V*_*n*_ will deduce whether the packet forwarding is successful or not, by overhearing the packet transmission from *V*_*n*+1_ to *V*_*n*+2_.

 (i) *Q*-*value update*: the updating of *Q*-value iterates at each node in each forwarding procedure. For 1-hop forwarding, at iteration *t*, node *i* ∈ *V*_*n*_ forwards a packet to *V*_*n*+1_ and nodes *i*′; neighbors of *i* in *V*_*n*_ monitor the packet forwarding. Then, *j* ∈ *V*_*n*+1_ is elected to continue packet forwarding. Therefore, node *i*updates its *Q*-values as
  (11) Qi′,it+1(sit,aft,amt)  =(1−α)Qi′, it(sit,aft,amt)   +α(rit+1(sit+1)+γ·ωsitV(sit)+γωsjtV(sjt)),

where *ω*_*s*_*i*_^*t*^_ and *ω*_*s*_*j*_^*t*^_ are, respectively, factors that weigh the total payoff in *V*_*n*_ and *V*_*n*+1_ and *V*(*s* ^*t*^) is the maximum payoff expressed by

(12) $$\begin{array}{c} V\left(s^{t}\right)=\underset{a_{f}^{t}}{\max}\underset{a_{m}^{t}}{\sum }\frac{C_{i^{\prime }}^{t}\left(s^{t},a_{m}^{t}\right)}{N\left(s\right)}Q_{i^{\prime },\;i}^{t}\left(s^{t},a_{f}^{t},a_{m}^{t}\right), \end{array}$$

where *C*_*i*′_^*t*^(*s* ^*t*^, *a*_*m*_^*t*^) counts the number of times agent *i* observed agent *i*′ taking action *a*_*m*_ in state *s* at packet *t* and *N*(*s*) is the total counts for all agents taking action *a*_*m*_ in state *s*. Therefore, *C*_*i*′_^*t*^(*s* ^*t*^, *a*_*m*_^*t*^)/*N*(*s*) is the probability in which the nodes other than *i* will select joint action *a*_*m*_ for packet *t* based on past experience.

So, for *i*′ ∈ *V*_*n*_ if agent *i*′ chooses *a*_*m*_ action, then

(13) $$\begin{array}{c} C_{i^{\prime }}^{t+1}\left(s^{t},a_{m}^{t}\right)=C_{i^{\prime }}^{t}\left(s^{t},a_{m}^{t}\right)+1,\\ N\left(s\right)=N\left(s\right)+1. \end{array}$$

Equation (11) shows that the *Q*-value of node *i* is a weighed sum of the *Q*-value of node *i* at the previous state, the action's immediate reward and the maximum payoff of the group *V*_*n*+1_ and the maximum payoff of the group *V*_*n*_.

 (i) *Reward function*: the reward function is defined as follows:

(14a)   ri=((∑j∈VnEj/NVn)−Ei)((∑j∈VnEj/NVn)−min⁡j∈Vn⁡Ej),

(14b) ri=RSSIi,Vn+1RSSIVn,Vn+1−σ·NVn.

Equation (14a) is used to calculate the reward when the packet forwarding is successful, where *E*_*i*_ represents the consumed energy for node *i* of the group *V*_*n*_. So, nodes with less energy consumption will receive positive rewards, and nodes with more energy consumption will receive negative rewards.

Equation (14b) is used to calculate the reward when the packet forwarding fails. The parameter *σ* takes 1 for the node that failed to forward data packet, whereas for the other nodes, it takes 0. So, the forwarding-node will receive a negative reward. The other nodes in *V*_*n*_ will receive positive reward according to their RSSI values.

In the opponent modeling case, all nodes in *V*_*n*_ are acting in a fully competitive task. So, the total sum of the attributed rewards to all cooperative nodes is zero.

After a certain number of iterations, nodes in *V*_*n*_ are able to use the learned policy to take appropriate actions.

### 2.5. Complexity Analysis

As noticed in the previous subsections, RL algorithms are composed of two main phases:

 (i) updating phase of the *Q*-values for each agent;
 (ii) node election for data forwarding.

For the *Q*-learning algorithm, the updating phase is realized through (4). The algorithm complexity concerning the *Q*-value updating is then equal to *N* ^2^.

For the node election phase, the node with the highest *Q*-value is elected for data forwarding:

(15) $$\begin{array}{c} a_{f}=\arg\underset{i}{\max}Q_{i}. \end{array}$$

So, the algorithm complexity concerning node election equals  *N*. Therefore, the algorithm complexity of the *Q*-learning algorithm equals to *N* + *N* ^2^.

For the opponent modeling algorithm, the updating phase is realized through (11). The algorithm complexity concerning the *Q*-value updating is then equal to *N* · (*N* − 1).

For the node election phase, the node with the highest payoff is elected for data forwarding:

(16) $$\begin{array}{c} a_{f}=\arg\underset{i}{\max}\underset{a_{m}^{t}}{\sum }\frac{C_{i^{\prime }}\left(s^{t},\;a_{m}^{t}\right)}{N\left(s\right)}Q_{i^{\prime },i}^{t}\left(s^{t},a_{f}^{t},a_{m}^{t}\right). \end{array}$$

So, the algorithm complexity concerning node election equals *N* ^2^. Therefore, the algorithm complexity of the *Q*-learning algorithm equals 2*N* ^2^ − *N*.

## 3. Performance Evaluation

### 3.1. Simulation Environment

For performance evaluation, we use TOSSIM simulation platform in order to evaluate parameters of interest such as energy consumption. TOSSIM is a discrete event simulator for TinyOS sensor networks that builds directly from the same TinyOS code written for the actual motes.

We simulate different topologies, sizes of WSN, and channel environment parameters (path loss and shadowing effects). The sink node is also placed in different positions. Simulation results concern network lifetime, packet delay (average delay to the sink, percentage of delayed packets, and percentage of lost packets), and energy consumption (network energy consumption and maximal energy consumption per node). Performance of RSSI/energy-CC algorithm is compared each time to MRL-CC algorithm.

The application of wildfire requires special measurement and transmission of temperature. Other parameters may be useful as moisture but are not considered in this paper. The amount of information transmitted is therefore likely to be low data rate. The area to cover, the forest, can be of different shapes. It can even be sparse. In this paper, we consider two different deployment architectures: uniform deployment and circular deployment.

In the forest environment, the transmission of information between different sensors can be significantly affected by the presence of trees. To evaluate the effect of this distortion on the quality of the proposed approach, we have also simulated the network in the presence of shadowing effect modeling this type of fading.

In Table 1, we give the parameters fixed for simulating the different versions of the algorithms.

### 3.2. Simulation Results

#### 3.2.1. Uniform Deployment

**Figure 4:**
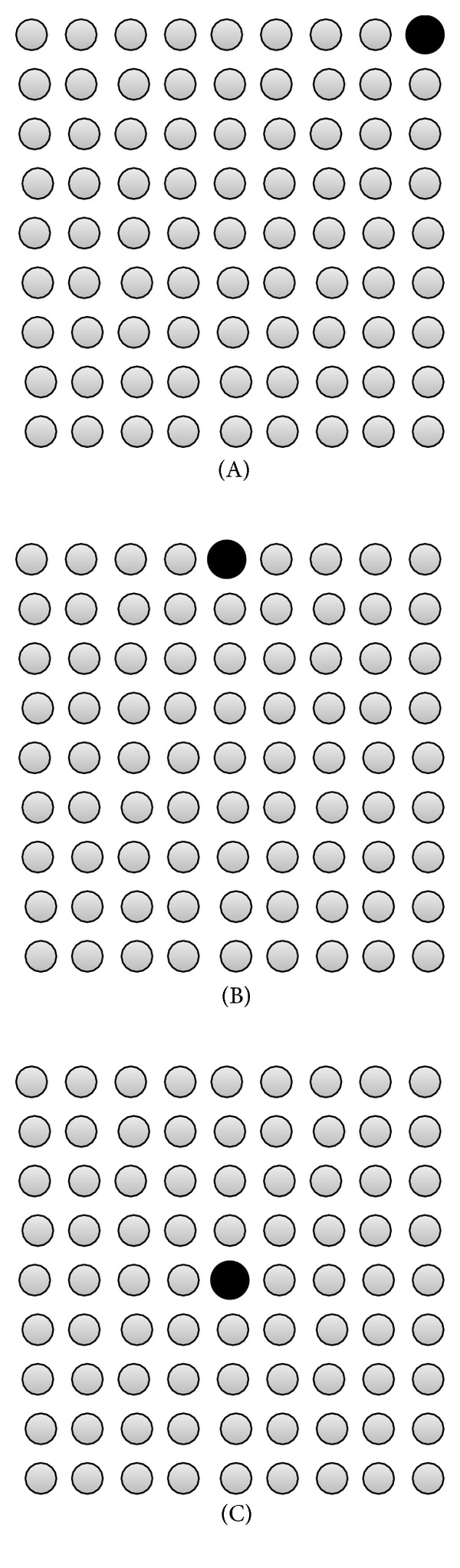
Sink node (in black) placement for topologies (A), (B), and (C).

We simulate a WSN where 81 sensor nodes are uniformly distributed in a 80 m × 80 m area (distance between 2 successive nodes is 10 m). The sink node is placed according to three different topologies (Figure 4).

**Figure 5:**
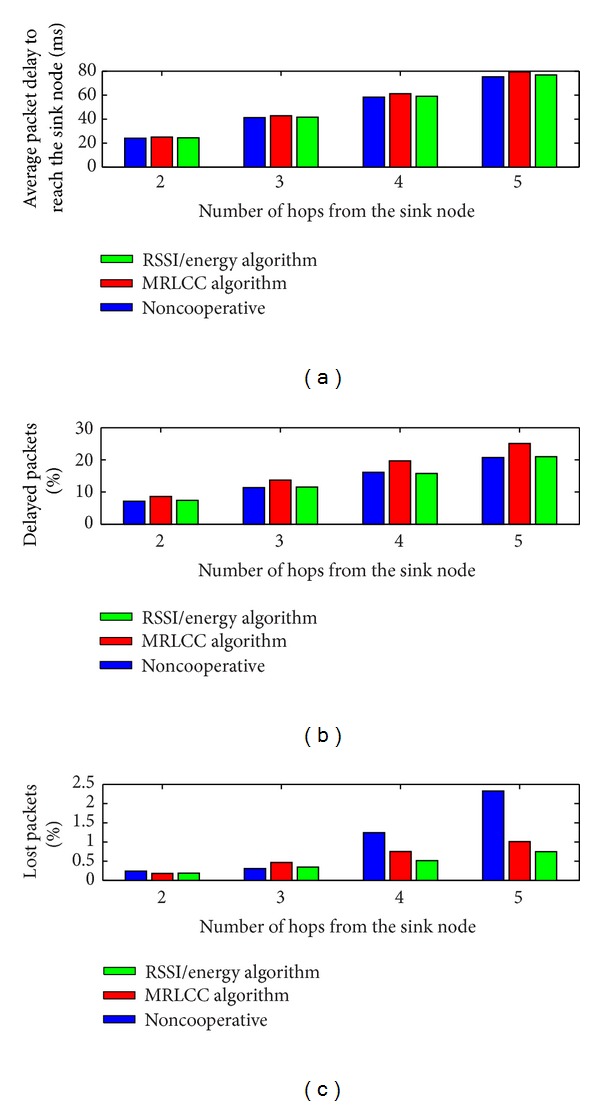
Average delay to the sink, percentage of delayed packets, and percentage of lost packets by averaging on the number of nodes being away with the same number of hops from the sink node.

*(a) Packet Delay Analysis.* We compute in Figure 5 the average delay to the sink, percentage of delayed packets, and percentage of lost packets.

The simulation results show that for noncooperative algorithm, the percentage of lost packets is huge compared to the MRL-CC algorithm and the RSSI/energy CC algorithm. However, in terms of percentage of delayed packets and average delay to the sink, the RSSI/energy-CC algorithm is lower than the MRL-CC algorithm. This is due to the fact that RSSI/energy-CC algorithm relies on the average link quality between the CN groups, which is performing at the same time in a competitive context. This competitive task allows a CN group to elect the node with the best RSSI for packet transmission.

**Figure 6:**
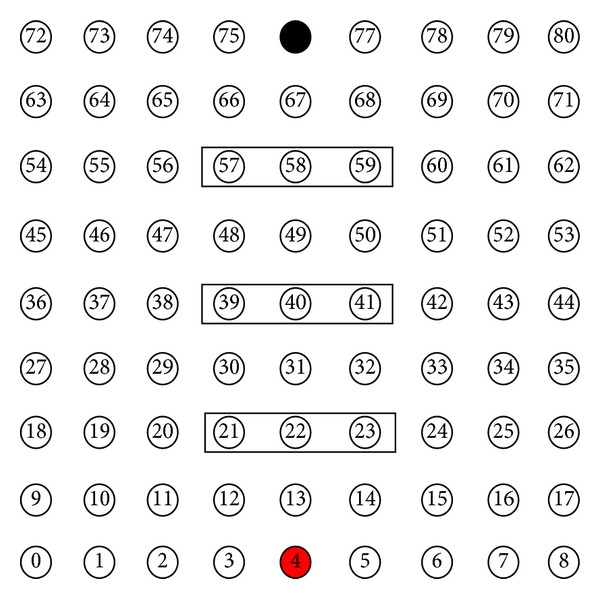
Selected CN groups for data transmission from source node 4 (in red) to sink node 76 (in black).

*(b) Energy Consumption in a Cooperative Node Group.* Figure 6 presents the selected CN groups for data transmission from node 4 to the sink node (topology B is considered).

**Figure 7:**
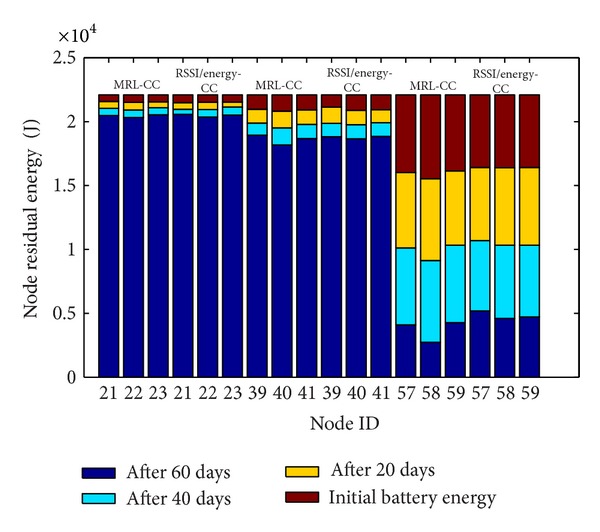
Energy consumption comparison for each selected CN group between MRL-CC algorithm and RSSI/energy-CC algorithm.

We display the residual battery energy for each selected CN group in Figure 7, and we compare energy consumption behavior between the MRL-CC algorithm and the RSSI/energy-CC algorithm.

Figure 7 shows that the behavior of energy consumption for each CN group is different when comparing MRL-CC algorithm and RSSI/energy-CC algorithm. For nodes which belong to the same CN group, the residual energy is more balanced for the RSSI/energy-CC algorithm. Thus, energy consumption is saved for each node in each CN group.

*(c) WSN Lifetime.* Network lifetime is defined as the time when the first node's battery is out of energy. For our case, we have compared the MRL-CC algorithm to the RSSI/energy-CC algorithm, computing at the same time the total energy consumed in the WSN (in J). Results are given in Table 2.

We also present in Table 3 the maximal lifetime during which all sensors can transmit to the sink node.

We can notice from Tables 2 and 3 that network lifetime is enhanced when comparing MRL-CC algorithm to RSSI/energy-CC algorithm. This enhancement is certainly due to some energy savings in the network.

**Figure 8:**
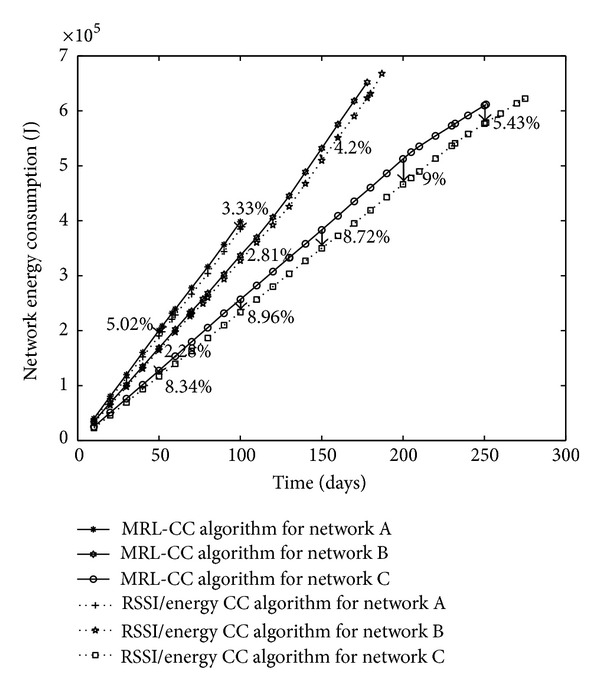
Network energy consumption, comparison between network architectures for MRL-CC and E/RSSI CC algorithm.

*(d) WSN Energy Consumption*. We first investigate energy consumption in the whole network. A comparison between the different network architectures for the two algorithms is presented in Figure 8.

Comparing network architectures, we conclude that C has the lowest energy consumption compared to A and B. So, network lifetime for C is the longest.

Simulation results also show that when comparing network energy consumption between the two algorithms for the same network architecture, network energy consumption is saved for the RSSI/energy CC algorithm compared to the MRL-CC algorithm. This is because the RSSI is considered for the decision of the node election for packet forwarding. Network energy consumption is saved from 3.33% to 5.19% for network A, from 2.28% to 6.23% for network B, and from 5.38% to 9.76% for network C.

**Figure 9:**
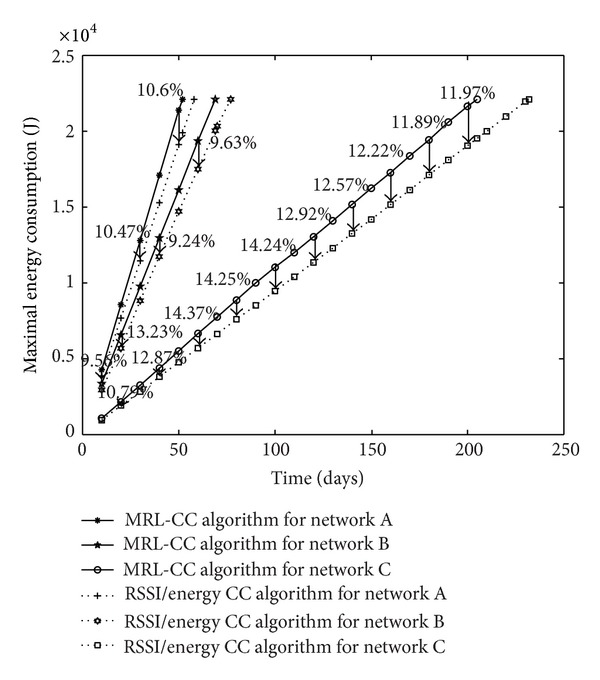
Maximal energy consumption in the whole WSN, comparison between MRL-CC and E/RSSI-CC algorithms for different network architectures.

At the same time, we compare the maximum energy consumption per node in the network, for the two algorithms. For each architecture, we obtain the charts presented in Figure 9.

The simulation results show that the maximum energy consumption per node is reduced for the RSSI/energy CC algorithm compared to MRL-CC algorithm. This is due to taking into account the energy consumption for the cooperative group before making the decision for node election. The maximal energy consumption is saved from 9.56% to 10.6% for network A, from 12.5% to 13.23% for network B, and from 10.79% to 14.76% for network C.

So, we can conclude that network lifetime enhancement is due to the enhancement of node's lifetime with maximal energy consumption.

In a second analysis of energy consumption, we propose to show results for extended grid networks where the sink is placed in the center (alike to topology C). Results about lifetime are shown in Tables 4 and 5.

We can notice from those tables that network lifetime is also enhanced for the RSSI/energy-CC algorithm.

**Figure 10:**
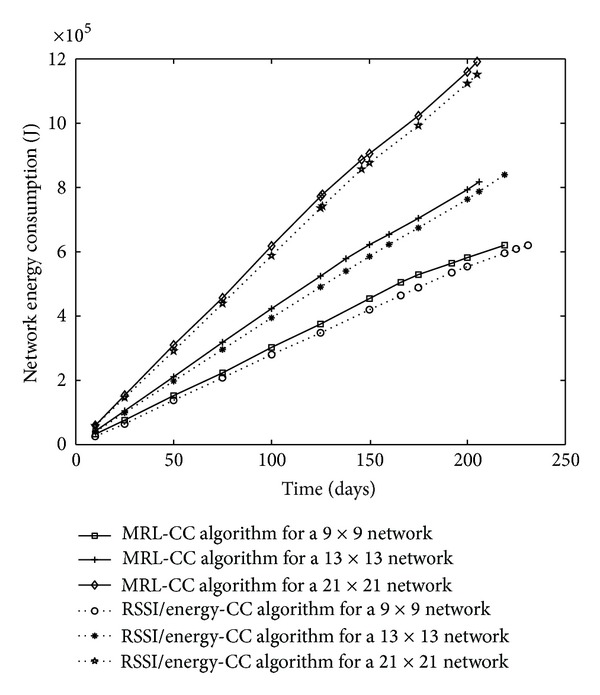
Network energy consumption, comparison between network architectures for MRL-CC and E/RSSI CC algorithms.

**Figure 11:**
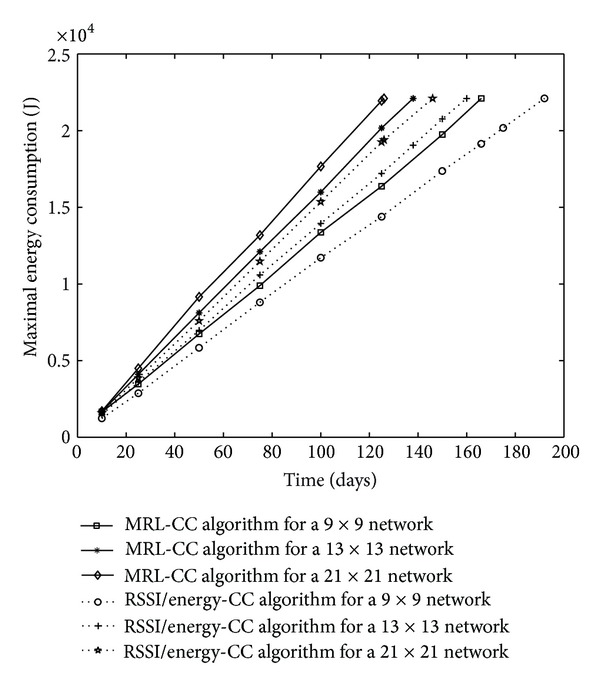
Maximal energy consumption in the whole WSN, comparison between MRL-CC and E/RSSI CC algorithms for different network architectures.

We also display results about network energy consumption in Figure 10, and the maximum energy consumption per node in the network in Figure 11.

Comparing network architecture, we conclude that 9 × 9 network has the lowest energy consumption compared to 13 × 13 and 21 × 21 networks. So, network lifetime for 9 × 9 network is the longest. Simulation results, in Figure 10, also show that when comparing network energy consumption between the two algorithms for the same network architecture, the network energy consumption is saved for the RSSI/energy CC algorithm compared to the MRL-CC algorithm. Network energy consumption is saved up to 9.49% for 9 × 9 network, up to 6.78% for 13 × 13 network, and up to 6.08% for 21 × 21 network.

In Figure 11, the simulation results show that the maximum energy per node is reduced for the RSSI/energy CC algorithm compared to MRL-CC algorithm. Thus, the maximal energy consumption is saved up to 17.17% for 9 × 9 network, up to 14.12% for 13 × 13 network, and up to 14.01% for 21 × 21 network.

#### 3.2.2. Energy Consumption for Circular Topology

**Figure 12:**
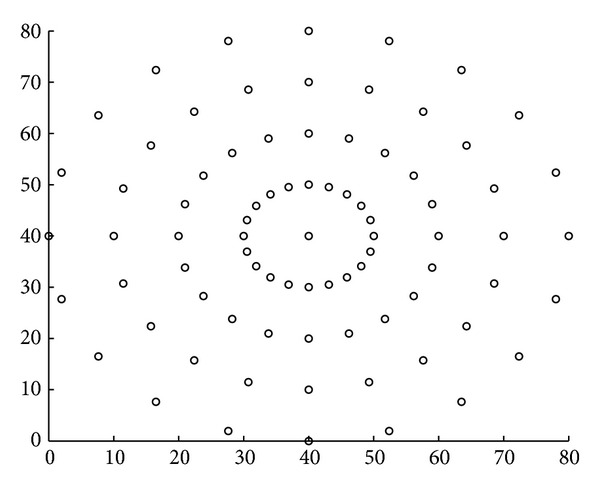
WSN topology in circles; sink node is at the center.

We also simulated our algorithms in the form of circles presented in Figure 12. The distance between circles is 10 meters.

**Figure 13:**
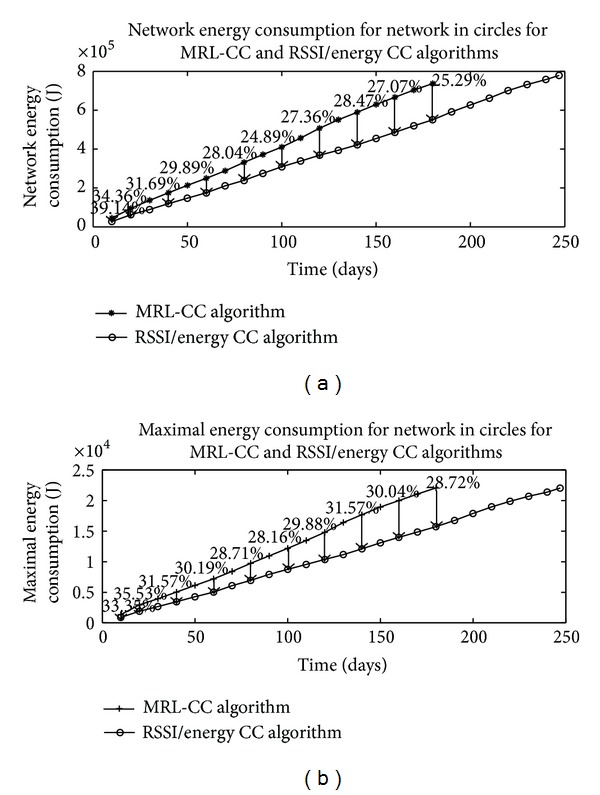
Network energy consumption and maximal energy consumption for network in form of circles, for MRL-CC algorithm and RSSI/energy CC algorithm.

Energy simulations for the network in circles for the two algorithms are presented in Figure 13.

The network lifetime for MRL-CC algorithm is 180 days. However, for the RSSI/energy CC algorithm, the network lifetime is 247 days. The gain in network lifetime is very valuable due to the special network topology. Network energy consumption savings go from 24.69% up to 39.14%. Also, for maximal energy consumption, savings are going from 28.16% up to 35.53%.

#### 3.2.3. Shadowing and Path-Loss Effect

**Figure 14:**
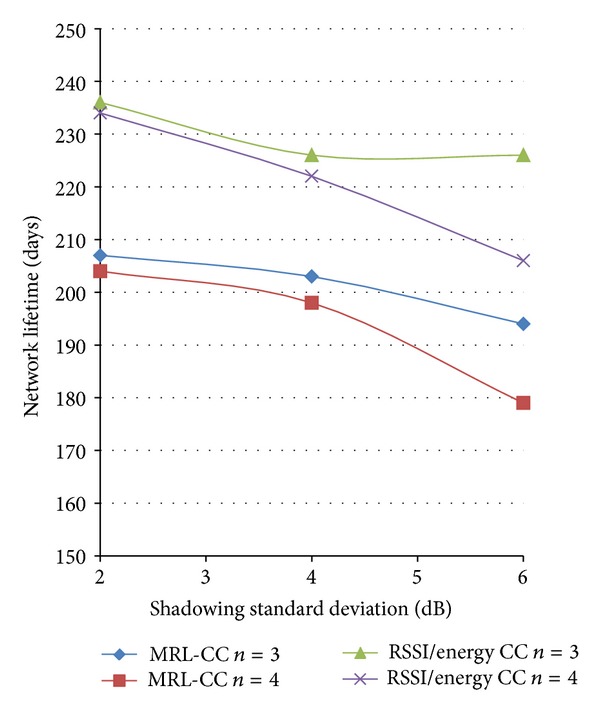
Network lifetime (architecture C) for different number of path losses, *n*, and different shadowing standard deviation, for the MRL-CC and the RSSI/energy algorithms.

We propose to use the network architecture C (uniform deployment) to simulate the network lifetime when path-loss number takes the values: *n* = 3 and 4, and shadowing deviation takes the values: *σ* = 2, 4 and 6 dB. Simulation results are shown in Figure 14.

 It is obviously clear that the network lifetime is reduced when the path-loss value increases and when the shadowing deviation increases. This result is both for the MRL-CC and the RSSI/energy CC algorithms. From that figure, we can also conclude that the RSSI/energy CC algorithm performs better than the MRL-CC algorithm in terms of network lifetime.

## 4. Conclusions

To help automatic monitoring of wildfire, we propose in this paper to deploy WSN. To design and optimize the routing protocol used for data aggregation in this network, we propose a new algorithm: the RSSI/energy-CC. This algorithm corresponds to the reinforcement learning optimization approach taking into account energy consumption and link quality measured by the RSSI, performing in a competitive task.

Simulations had shown that this algorithm is efficient in terms of percentage of lost packets, network energy consumption, maximal energy consumption per node, and network lifetime.

In future research, we will consider both the case of multiple sinks in the WSN in order to better process network energy consumption and better enhance the network lifetime and sparse deployment which describes better the forest environment.

## Figures and Tables

**Table 1:** Simulation parameters.

Packet delivery	every 200 milliseconds
Packet size	17 Bytes
Reforwarding time	10 ms
Communication range	30 m
Initial battery energy	2 Li-ion AA batteries
Path-loss exponent	2
Shadowing standard deviation	2 dB
MAC object	CSMA protocol
Node used for simulation	Mica2 platform

**Table 2:** Network lifetime (in days) till the first node dies.

Network architecture	A	B	C
MRL-CC	52	69	205
RSSI/energy CC	58	77	232

**Table 3:** Network lifetime (in days) till the WSN cannot transmit to the sink node.

Network architecture	A	B	C
MRL-CC	100	178	251
RSSI/energy CC	101	187	275

**Table 4:** Network lifetime (in days) till the first node dies.

Network architecture	9 × 9	13 × 13	21 × 21
MRL-CC	166	138	126
RSSI/energy CC	192	160	146

**Table 5:** Network lifetime (in days) till the WSN can not transmit to the sink node.

Network architecture	9 × 9	13 × 13	21 × 21
MRL-CC	219	206	205
RSSI/energy CC	231	219	205
